# The complete chloroplast genome sequence of *Hemerocallis minor* (Asphodelaceae)

**DOI:** 10.1080/23802359.2022.2093663

**Published:** 2022-07-08

**Authors:** Xiaofei Zhang, Lixin Lang, Xuwen Shang, Zhenting Wang, Lanling Jiang, Xinhui Pei, Jiaojiao Lu, Dan Li, Jiaming Yang

**Affiliations:** a Institute of Floriculture, Liaoning Academy of Agricultural Sciences, Shenyang, China; b China Construction Third Bureau, Urban Construction Co. Ltd, Shenyang, China; c Shenyang Agricultural University, Shenyang, China

**Keywords:** Chloroplast genome, *Hemerocallis minor*, phylogenetic analysis

## Abstract

*Hemerocallis minor* is a kind of wild plant with high ornamental value. In this study, we sequenced the complete chloroplast genome of *H. minor* by using Illumina sequencing techniques. The whole chloroplast genome was 156,063 bp in size, consisting of a large single-copy (LSC) region of 84,820 bp, a small single-copy (SSC) region of 18,505 bp, and a pair of inverted repeats (IRa and IRb) regions of 26,369 bp. The chloroplast genome contained 134 genes in total, including 88 protein-coding genes, 38 tRNA genes, and eight rRNA genes. The overall GC content was 37.34%. Phylogenetic analysis showed that *H. minor* was closely related to *Hemerocallis citrina* of the same genus.

*Hemerocallis minor*, belonging to Asphodelaceae family (Zhang 2020), is born in grassland, hillside or forest below the elevation of 2300 m, and is widely distributed in northern China, Korea and the Soviet Union (Chen and Junko 2000). *H. minor* germinates in early spring and has high ornamental value due to its emerald green and lush leaves as well as gorgeous and beautiful flowers (Qin and Shang 2002). At the same time, *H. minor* has a wide range of pharmacological effects, including anti-tumor, antibacterial, inhibition of vascular smooth muscle cell proliferation, diuresis, effect on the immune system, anti-inflammatory and other effects, and also has a significant effect on depression (Zhang 2017). So far, researchers have paid so little attention to *H. minor* that it has been rarely literature reported. Lack of genetic and genomic information further limits better development and utilization of *H. minor*. Based on the characteristics of small genome, simple structure, and highly conservative sequence of chloroplasts, chloroplast genome is increasingly becoming a new method to study the biological problems of Hemerocallis (https://doi.org/10.1080/23802359.2020.1726227). Therefore, we reported the complete chloroplast genome sequence of *H. minor* and revealed its phylogenetic relationship with related species in the Asphodelaceae.

The fresh leaf sample of *H. minor* was collected from the Germplasm Resource Garden of Institute of Floriculture, Liaoning Academy of Agricultural Sciences (41°49′14″N, 123°32′47″E), Shenyang city, Liaoning province, China. A specimen was deposited at Institute of Floriculture of Liaoning Academy of Agricultural Sciences (Xiaofei Zhang and 1249308231@qq.com) under the voucher number ASPH_HEM_MIN_01. The complete chloroplast genome DNA was extracted using the improved CTAB method (Doyle 1987; Yang et al. 2014), and sequenced on the Illumina HiSep2500 sequencing platform with 150 bp paired-end library by Nanjing Jisi Huiyuan Biological Technology Co. Ltd. (Nanjing, China). Clean reads were filtered and assembled using SPAdes software (Bankevich et al. 2012) and annotated using Geneious Prime (www.geneious.com) with *Hemerocallis citrina* (MN872235.1) as the reference to a complete chloroplast genome and submitted to GenBank (accession number MW845762.1).

The complete chloroplast genome of *H. minor* was 156,063 bp in length and had a typical quadripartite structure, consisting of a large single-copy (LSC) region of 84,820 bp, a small single-copy (SSC) region of 18,505 bp, and two inverted repeat (IRa and IRb) regions of 26,369 bp. The chloroplast genome contained 134 genes, including 88 protein-coding genes, 38 tRNA genes, and eight rRNA genes. The overall GC content of the whole plastome was 37.34%, whereas the corresponding values of the LSC, SSC, and IR regions were 35.07%, 31.98%, and 42.86%, respectively. Two hundred and thirty-one SSRs were detected by using Perl script MISA (Thiel et al. 2003) in the whole chloroplast genome.

To investigate phylogeny of *H. minor*, a total of nine chloroplast genome sequences from related species in Asphodelaceae and *Hosta plantaginea* (Asparagaceae) was used as an outgroup were downloaded from the NCBI database to construct the phylogenetic trees by neighbor-joining method of protein coding genes that obtained from a maximum-likelihood analysis with 1000 bootstraps using MEGA7 (Kumar et al. 2016). The result showed that *H. minor* was closely related to *H. citrina* of the same genus. This study would be accordingly beneficial to potential studies on phylogenetics of the genus and related group in Asphodelaceae (Figure 1).

**Figure 1.**
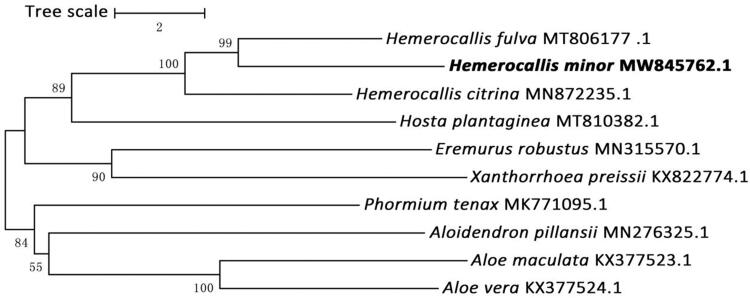
Phylogenetic tree was constructed based on nine complete chloroplast genome sequences of Asphodelaceae and *Hosta plantaginea* (Asparagaceae), and all the sequences were downloaded from NCBI GenBank.

## Permission statement

Collection on *H. minor* in the article, it fully complies with ‘Regulations of the People's Republic of China on the Protection of Wild Plants’ and the guidelines of the Liaoning Academy of Agricultural Sciences.

## Data Availability

The genome sequence data that support the findings of this study are openly available in GenBank of NCBI at https://www.ncbi.nlm.nih.gov/nuccore/MW845762 under the accession no. MW845762.1. The associated BioProject, SRA, and Bio-Sample numbers are PRJNA753232, SRR15420788, and SAMN20691849, respectively.
